# Non-cognitive skills and social gaps in digital skills: Evidence from ICILS 2018^☆^

**DOI:** 10.1016/j.lindif.2022.102254

**Published:** 2023-02

**Authors:** Zbigniew Karpiński, Giorgio Di Pietro, Federico Biagi

**Affiliations:** a European Commission Joint Research Centre, Edificio EXPO, calle Inca Garcilaso 3, 41092 Seville, Spain; b European Commission Joint Research Centre, Building 26A CCR, via Enrico Fermi 2749, 21027 Ispra, Italy; c Institute of Philosophy and Sociology Polish Academy of Sciences, ul. Nowy Swiat 72, 00-330 Warsaw, Poland; d IZA, Schaumburg-Lippe-Strasse 5-9, 53113 Bonn, Germany

**Keywords:** Computer and Information Literacy (CIL), ICILS 2018, Non-cognitive skills, Gender, Socioeconomic, Native-immigrant gaps in CIL

## Abstract

Using data from the 2018 round of the International Computer and Literacy Survey (ICILS), this study looks at the effect of non-cognitive skills (e.g., motivation, ambition, and conscientiousness) on digital competences as measured by the Computer and Information Literacy (CIL) test score. Non-cognitive skills may be especially important in low-stakes tests such as ICILS, where students face no consequences — positive or negative — as a result of their performance. The empirical results show that several non-self-reported measures acting as proxies for non-cognitive skills are significant determinants of CIL test scores. Furthermore, the findings point at differences in non-cognitive skills across gender, immigrant background, and socioeconomic status. This suggests that one should be cautious when inferring about inequality in digital competences along these dimensions using low-stakes test scores, and underscores the importance of controlling for non-cognitive skills.

## Highlights

• ICILS assesses 8th-grade students' digital skills.
• Students' computer and information literacy score is used.
• Digital skills are found to depend on non-cognitive skills (e.g., conscientiousness).
• Non-cognitive skills partially explain the gender and socioeconomic gaps in digital skills.

## 1 Introduction

Results from large-scale assessments (e.g., PISA, TIMSS, PIRLS) are used for international benchmarking and institutional accountability (Zilberberg et al., 2014). They have also triggered educational policy reforms in several countries (Choi & Jerrim, 2016; Dobbins & Martens, 2012). The rationale behind this is that these test scores — capturing students' knowledge and problem-solving skills in different domains — can be used to compare the efficacy and equity of educational systems around the world. However, several studies (Borghans et al., 2008; Hawthorne et al., 2015) suggest that one needs to be cautious in drawing strong conclusions from such tests as, along with students' cognitive skills, they also reflect their willingness to perform well in the absence of extrinsic reward. Such argument is also supported by the findings of Gneezy et al. (2019) showing that students in various countries come to these assessment tests with different incentives to do well.

Existing research shows that students' performance on a cognitive test depends not only on the actual cognitive abilities they possess, but also on their non-cognitive skills.^1^ While cognitive and non-cognitive skills are correlated, they capture different dimensions with separate effects on students' outcomes (Duckworth et al., 2007; Heckman et al., 2019). Non-cognitive skills have been defined as personality traits that influence the way pupils think, feel and behave (Borghans et al., 2008). Motivation, perseverance, self-control, conscientiousness,^2^ grit^3^ as well as resilience, creativity, teamwork and social competencies are examples of non-cognitive skills that are highly important for student achievement and predictive of academic success (Gutman & Schoon, 2013; Kattan, 2017; Khine & Areepattamannil, 2016).

Non-cognitive skills may influence student performance particularly on low-stakes tests (i.e., tests that have no impact on students' grades or other academic achievements), such as the international large-scale assessments. While some students may be motivated to perform well irrespective of the rewards offered, the lack of academic consequences for test-takers may discourage others from exerting the maximal effort.

We use data from the 2018 wave of the International Computer and Information Literacy Survey (ICILS), a large-scale, international assessment of 8^th^-grade students' digital competences. We focus on the Computer and Information Literacy (CIL) test, which is expected to reflect “individual's ability to use computers to investigate, create, and communicate in order to participate effectively at home, at school, in the workplace, and in society” (Fraillon et al., 2020b, p. 16). As other large-scale assessments in education, ICILS is a low-stakes test and uses a task-based approach to measuring students' digital skills. Task-based measures of digital skills are preferred over measures based on self-reports, given that the latter are subject to biases and misrepresentations (Juhaňák et al., 2019).

Following recent literature (e.g., Balart et al., 2018; Zamarro et al., 2019), we construct three non-self-reported^4^ measures that act as proxies for students' non-cognitive skills. They are derived from patterns of responses to the CIL test and to the background questionnaire. Specifically, these proxies are: (*a*) drop in response time to test questions; (*b*) drop in performance along the test; (*c*) non-response rate to the background questionnaire. As observed by Borghans and Schils (2018), differences in performance drop during the test are associated with non-cognitive skills such as motivation and ambition. The decline in performance on later portions of the PISA test found in both Borgonovi and Biecek (2016) and Zamarro et al. (2019) is linked to lower student motivation (see Borgonovi & Biecek, 2016; Zamarro et al., 2019). Using information from the Programme for the International Assessment of Adult Competencies (PIAAC), Anghel and Balart (2017) argue that time per question is a good indicator for non-cognitive skills such as motivation, ability to stay focused or grit/thrift.^5^ Hedengren and Stratmann (2012) and Hitt et al. (2016) show that non-response to non-challenging questions in surveys is negatively correlated with traits such as conscientiousness and perseverance (see also Zamarro et al., 2020).

This study addresses the following research questions: (1) Do our non-self-reported proxies for students' non-cognitive skills affect student digital competences as measured by the CIL scores? (2) To what extent and in which direction do digital competence gaps along the lines of gender, socioeconomic background, and immigrant status change after accounting for the effect of our non-cognitive skill proxies? The answers to these questions add to the existing literature in two ways. First, our analysis extends the results of earlier studies (e.g., Borghans & Schils, 2018) — which used similar proxies to examine the impact of non-cognitive skills on mathematics, reading, and science test scores — to the area of digital literacy. Indirect evidence suggests that non-cognitive skills may play an important role in predicting ICT-related outcomes. For instance, Grundke et al. (2018) find that digital-intensive industries reward especially workers with high levels of self-organisation (see also Morandini et al., 2020). Second, while data from ICILS 2018 show that females, students from higher socioeconomic status and native students have higher CIL test scores (Fraillon et al., 2020b), we explore the role of unobserved variables such as non-cognitive skills in explaining these associations. Several scholars argue that this research is much needed. For instance, in their recent meta-analysis, Scherer and Siddiq (2019) emphasise the importance of exploring additional confounding variables behind the relationship between socioeconomic status and digital skills. The present study addresses precisely this need by looking at the potential contribution of non-cognitive skills.

## 2 Background and hypotheses

In this section, we first review studies on achievement and non-cognitive skills, focusing on the impact of those non-cognitive skills that the proxies employed in this paper are expected to capture (i.e., motivation, ambition, self-control, ability to stay focused, grit, agreeableness, need for achievement, perseverance, and conscientiousness). In the second part, we look at the literature addressing gaps in digital skills along gender, socioeconomic status, and immigrant background lines and go on to hypothesise how these gaps change when non-cognitive skills are accounted for.

### 2.1 Non cognitive skills and achievement

Many studies have analysed the importance of non-cognitive traits in student achievement. Lechner et al. (2019) find that non-cognitive skills contribute to the educational outcomes of children and adolescents above and beyond cognitive skills and socioeconomic background. Lee (2020) attempts to identify the most important non-cognitive predictors of student achievement. While Palacios-Abad (2021) concludes that student effort influences the likelihood of completing higher education, Zamarro et al. (2019), using PISA data, demonstrate that this factor explains a considerable part of the variation in test scores across countries.

Moving on to the impact of the specific non-cognitive skills that our proxies attempt to account for, Zigler and Butterfield (1968) provide evidence that motivation significantly improves performance on IQ tests. Similarly, Borghans et al. (2008) find that people with higher motivation generally perform better on cognitive tests. Conscientiousness is also strongly related to academic performance as measured by final grades (Conrad & Patry, 2012) or test scores in mathematics (Meyer et al., 2019).

Tangney et al. (2004) find that students with higher self-control have higher GPAs. Duckworth et al. (2010) conclude that self-control causally impacts academic performance: they find that changes in self-control over time predict variations in GPA.

Using individual-level data from a US university, Duckworth et al. (2007) find that grit scores are associated with higher GPAs and such positive relationship turns out to be robust even when SAT scores are controlled for. This result is in line with that of Strayhorn (2014) who shows that grit influences grades. Hagger and Hamilton (2019) observe that grit is a predictor of science grades in a sample of secondary school students. Thorsen et al. (2021) and Xu et al. (2021) find that perseverance, too, is positively associated with student achievement.

Finally, studies conducted in UK Farsides and Woodfield (2003) and US Bidjerano and Dai (2007) found that agreeableness is positively related to final grades and GPA, respectively.

### 2.2 Social drivers of digital skills inequalities and the role of non-cognitive skills

#### 2.2.1 The gender dimension

The magnitude and direction of gender differences in digital competencies depends to a large extent on the type of digital skill that is being evaluated. Various studies using data from the first round of ICILS find that female students outperform male students on the CIL test (Gebhardt et al., 2019; Hatlevik et al., 2018; Punter et al., 2017). Upon a closer inspection of the data, Gebhardt et al. (2019) conclude that female students scored higher on tasks involving communication and creativity, while male students scored higher on tasks requiring some technical skills (see also Guggemos, 2021). Gender gaps in digital skills favouring girls are also found by Aesaert and van Braak (2015) in their study of Flemish grade 6 students. In addition, meta-analysis results by Siddiq and Scherer (2019) indicate a positive and statistically significant gap in digital skills in favour of female students. Using a large longitudinal sample of German students, Gnambs (2021) tracks changes in their digital skills and digital confidence over the course of three years, i.e., between the ages of 15 and 18. He shows that, while gender gaps favouring male students were initially negligible, they grew considerably over the period under study.

As for non-cognitive skills, several studies indicate that, on average, women show higher motivation, especially in low-stakes tests. DeMars et al. (2013) show consistent evidence supporting the proposition that men exhibit lower test-taking motivation than women (Di Chiacchio et al., 2016; Yunus & Ali, 2009, see also). Additionally, female students score higher on both conscientiousness (Schmitt et al., 2008) and agreeableness (Costa et al., 2001; Feingold, 1994). Wehner and Schils (2021) argue that conscientiousness levels tend to be higher among females relative to males, particularly with respect to aspects such as better organisation and diligence. Based on data from a nationally representative sample of American students, DiPrete and Jennings (2012) find that teachers consistently rate girls higher than boys on various non-cognitive skills, including self-control. Using data from PISA 2012, Arellano et al. (2018) look at the effect of non-cognitive skills on test scores. They find that, while females have higher scores than males, accounting for perseverance and motivation reduces the gender gap.

In light of the results of previous studies, the following hypothesis is made:

Hypothesis 1 *Assuming that girls outperform boys on the CIL test, accounting for differences in non-cognitive skills between the former and the latter reduces the size of the gender gap in the CIL scores.*

#### 2.2.2 The socioeconomic dimension

Existing studies clearly indicate that individuals from more advantaged backgrounds are more likely to be digitally skilled, on average, than those from less advantaged backgrounds. This finding seems to hold regardless of the specific variable representing socioeconomic status (mother's education, see Aesaert & van Braak, 2015; a composite index based on parental education, parental occupation, and household possessions, see Hatlevik et al., 2018; cultural capital, see Hatlevik, Guðmundsdóttir, et al., 2015; Hatlevik, Ottestad, et al., 2015).

There is also a large consensus that socioeconomic status is an important determinant of students' non-cognitive skills (see, for example, Liu, 2019) and especially motivation. Children from higher status families are more likely to develop an academic intrinsic motivation than those from lower status families (Farkas, 2003). Two reasons are often advanced in an attempt to explain such finding. First, parents' beliefs and expectations may exert a significant influence on their children's intrinsic motivation (Bandura, 1997). Less educated parents are less likely to place a high value on their children's education and hence are less inclined to transmit them a positive attitude towards learning and their academic abilities (Gottfried et al., 1994; Wang et al., 2020). As a result of this, students from more advantaged backgrounds are more likely to exhibit an autonomous learning behaviour than their peers from less advantaged backgrounds (Kormos & Kiddle, 2013). Kormos and Csizér (2014) indicate that autonomous learning and effective self-regulated strategies are related to student motivation. Second, socioeconomic status may influence the type of activities children do outside home. For instance, children of more educated parents are more likely to participate in extracurricular activities that stimulate academic mindset and motivation (Usher & Kober, 2012). Using 2015 PISA data, Azzolini et al. (2019) examine the relationship between student perseverance during the test and socioeconomic status. They find that students from advantaged backgrounds have more perseverance than their less advantaged peers.

Based on these findings, the following hypothesis is proposed:

Hypothesis 2 *Assuming that students from more advantaged backgrounds outperform students from less advantaged backgrounds on the CIL test, accounting for differences in non-cognitive skills between the former and the latter reduces the size of the socioeconomic gap in the CIL scores.*

#### 2.2.3 The immigrant background dimension

Immigrant students have, on average, lower academic achievements, on than their native peers. This holds in general and also for digital skills (Fraillon et al., 2020b, p. 83). In spite of this, students with an immigrant background tend to report higher levels of motivation (Alivernini et al., 2018; Areepattamannil & Freeman, 2008; Schleicher, 2006). Two explanations can be proposed. First, the higher academic motivation of immigrant students may stem from a sense of family obligation (Fuligni & Tseng, 1999). They may feel obliged to do well at school in order not to disappoint their parents (Urdan, 2004), many of whom have made enormous sacrifices by leaving their country of origin in search for a better life for themselves and their children. Educational success is perceived by these students as a way to repay their parents for their sacrifices and investment and financially assist their families in the future (Tseng, 2002). Second, it is also possible that students from immigrant backgrounds are autonomously motivated to learn (Alivernini et al., 2018). They are aware that education is an important asset that will help integrate them successfully into the host country both economically and sociallly (St-Hilaire, 2002). Individuals with a higher level of education have better job opportunities and higher incomes. Education also plays a crucial role in helping immigrants assimilate the culture of the host country.

Using data on 11^th^-grade and 12^th^-grade students from two public secondary schools in Great Toronto Area, Areepattamannil and Freeman (2008) find that immigrant adolescents report higher intrinsic and extrinsic motivation in comparison with their non-immigrant counterparts. Schleicher (2006) uses PISA 2003 data to look at differences in learning dispositions between immigrant students and their native peers. He finds that the former report higher levels of motivation in mathematics than the latter. Employing data from a representative sample of Italian 5^th^-grade students, not only do Alivernini et al. (2018) find that immigrant students are more academically motivated than natives, but they also conclude that first generation immigrant pupils are more motivated and engaged in the school context than their second generation counterparts.

Our final hypothesis is:

Hypothesis 3 *Assuming that foreign-born students fare worse on the CIL test than their native-born peers, accounting for differences in non-cognitive skills between the former and the latter widens the immigrant-native gap in the CIL scores.*

## 3 Data and variables

This study uses data from the second round of the ICILS survey, conducted in 2018. While most ICILS participants are countries, a city (i.e., Moscow) and a region (i.e., North Rhine-Westphalia) have also taken part in this survey. The sample is designed to be representative of the relevant population of 8^th^-grade students. While, in addition to students, ICILS collects also information on teachers and headmasters related to the schools attended by the students, these data are not used in our analysis.

Overall, sample sizes of student participants in the second round of ICILS range from 1991 in North Rhine-Westphalia to 6790 in the United States, and median sample size is equal to 2908. However, due to missing values on key variables, which we listwise-deleted, the analysis is based on somewhat reduced samples. The number of missing cases varies by country/region/city, ranging from 112 in Denmark to 1394 in Luxembourg. The dataset that we finally use for all the analyses reported in this paper comprises 40403 students. All individuals whose data were collected in the survey used in this study provided consent for publication of these data (Fraillon et al., 2020a, chap. 8).

We use the following student characteristics as independent variables in our analysis: gender (coded 1 for female, and 0 for male), age (in years), immigrant background (captured by a binary variable that takes the value of 1 if both parents were born abroad, and 0 if at least one parent was born in the country of the test), the number of computers (i.e., desktop computers or laptops) at home (captured by a binary variable that takes the value of 1 if there are at least two computers, and 0 otherwise), experience using computers (dummy variables are created for the following categories: never or less than one year; at least 1 year but less than 3 years; at least 3 years but less than 5 years; at least 5 years but less than 7 years; and 7 years or more), parental education (captured by a binary variable coded 1 for students who have at least one parent with tertiary education, and 0 otherwise). Note that the information on all the student characteristics, including parental education, is self-reported by students.

### 3.1 The design of the ICILS test

An important feature of the ICILS test lies in its “rotated design”: different versions of the test are developed and then allocated randomly among participants. The versions differ in terms of content (i.e., some tasks are included in some versions, but not in others) and ordering of the tasks (i.e., the same task can be placed early or late in the test question sequence, depending on the version). This latter characteristic allows for studying the effect of item position, which is exogenously varied, on students' behaviour (e.g., response time or the probability of answering correctly).

Digital skills are a latent ability that is not directly observable and is estimated with considerable uncertainty. To acknowledge the uncertainty, students are assigned a set of five “plausible values” to represent their performance on the test (Khorramdel et al., 2020; Lechner et al., 2021). These plausible values are scaled to have a mean of 500 and a standard deviation of 100. Analyses are performed on each plausible value separately and the results of these analyses are then aggregated following Rubin's rules (Drechsler, 2020) to generate point estimates of the parameters of interest and their standard errors. Table 1 shows the average scores on the CIL test, along with the corresponding standard errors, for each country/region/city participating in ICILS 2018. Students' performance on the test, as represented by the plausible values, is our dependent variable in the analysis.

**Table 1:** Average CIL test scores, standard deviations and sample sizes, by participating country/region/city.

Country/region/city	Mean	S.D.	Sample size
Denmark	552.6	65.5	2404
Moscow	549.1	68.0	2852
Korea	542.1	94.9	2875
Finland	530.7	80.7	2546
United States	518.9	80.7	6790
Germany	518.3	80.1	3655
Portugal	516.5	71.1	3221
North Rhine-Westphalia	514.7	75.1	1991
France	498.7	80.5	2940
Luxembourg	481.8	83.7	5401
Chile	475.9	83.3	3092
Italy	461.0	81.7	2810
Uruguay	450.4	100.5	2613
Kazakhstan	395.2	106.0	3371

Before presenting the results of the analysis, it is important to understand how the ICILS test was organised. Overall, five different CIL modules were developed, each lasting up to 30 min. An individual test comprised two of these modules, so that a total of 20 different versions of the CIL test could be obtained. These versions were allocated randomly to students. They had an hour to complete the test, after which they filled in the background questionnaire.

### 3.2 Proxies for non-cognitive skills

Along with the student characteristics described above, this study uses three proxies for non-cognitive skills. The first proxy is the drop in response time to successive questions in the test. As mentioned in the introduction, the average time per question is indicative of such non-cognitive skills as the ability to stay focused or grit (Anghel & Balart, 2017). By implication, a decline in the response time over the course of the test can be interpreted in terms of lack of these skills. Given the rotated test design, we can measure a decline in the response time during the test net of the characteristics of the questions (e.g., difficulty or complexity).

To measure drop in response time, we adapt the method developed by Borghans and Schils (2018). We assign a sequence number to each question in a given version of the test and go on to rescale the numbers so that the first question has number 0 and the last question has number 1. Then we regress response time on the rescaled question/sequence number. The coefficient for the latter variable represents the average difference in response time between the last and the first question on the test, regardless of the test version. We use mixed-effect linear regression with crossed random effects associated with respondents and with questions to account for a “grouped” character of our data: each student answers more than one question and the same question is answered by more than one student, so the observations can be said to be “nested within” respondents and test questions. By fitting a mixed-effect model with varying slopes, we can extract student-specific slopes from the model and use them as a variable representing drop in response time.

Our second proxy of non-cognitive skills is the drop in the probability of a correct answer to each successive question on the test. Following the literature reviewed above, this variable is associated with such non-cognitive skills as self-control (Borgonovi & Biecek, 2016), motivation and ambition (Borghans & Schils, 2018), conscientiousness, agreeableness and the need for achievement (Borghans & Schils, 2018). Our approach to measuring performance drop consists in modelling the probability of a correct answer to a given question as a function of its placement in the test.

Building on the original work by Borghans and Schils (2018), we retrieve performance drop from students' responses to test questions while following the same steps described above for drop in response times. We regress responses to the test questions on the (rescaled) question/sequence number using mixed-effect linear model with crossed random effects associated with respondents and with questions. Correct responses are coded as 1 and incorrect responses as 0. Responses with partial credit are coded as 0.5. We fit a random-intercept and random-slope model, where the slope is allowed to vary across students, and then we extract student-specific slopes to use them as proxies for the above-mentioned non-cognitive skills. The slope coefficient for the (rescaled) question number is the difference in the probability of a correct response between the last and the first question in the test. Again, because of the rotated test design used in ICILS, the probability is estimated net of characteristics of the questions which also affect the probability of a correct response. Negative values of the variable indicate lower levels of the non-cognitive skills that this variable represents, while its positive values suggest higher levels of those skills.

Our third proxy for non-cognitive skills derives from counting the number of times students provide meaningful responses to questions in the background questionnaire. In some cases, students leave out a question without answering. Non-response, or a failure to answer questions in the background questionnaire can indicate a low level of conscientiousness (Hedengren & Stratmann, 2012; Hitt et al., 2016; Soland et al., 2019). Subtracting the number of unanswered questions from the total number of questions in the background questionnaire gives us the number of meaningful responses provided by a student. By implication, this number is expected to be positively associated with conscientiousness. For the ease of interpretation, we divide it by the total number of questions, so that the resulting values are bounded between 0 and 1, with higher values indicating higher levels of conscientiousness. In the text, we refer to this variable as “item response rate”.

In Table 2, we show pairwise correlations between the variables used in the current study. In most cases, correlations are rather moderate. Specifically, the correlations between our three proxies for non-cognitive skills are not very strong. This confirms that, rather than reflecting the same underlying construct, the three variables represent distinct and separate constructs.

**Table 2:** Pairwise correlations between the variables used in the study; ICILS 2018, pooled sample. NB: For computer experience, the reported values refer to Kendall's *τ* rank correlation coefficient.

	Female	Age	Migrant	Computer experience	Computers at home	Parent with higher education	Time drop	Performance drop	Response rate	CIL test score
Female		−0.06^⁎⁎⁎^	0	0.01^⁎^	0.02^⁎⁎^	−0.03^⁎⁎⁎^	0.13^⁎⁎⁎^	0.11^⁎⁎⁎^	0.05^⁎⁎⁎^	0.12^⁎⁎⁎^
Age			0.04^⁎⁎⁎^	0.06^⁎⁎⁎^	−0.03^⁎⁎⁎^	0	−0.09^⁎⁎⁎^	−0.02^⁎⁎^	−0.03^⁎⁎⁎^	−0.02
Migrant				−0.02^⁎^	−0.04^⁎⁎⁎^	−0.04^⁎⁎⁎^	0	−0.04^⁎⁎⁎^	−0.05^⁎⁎⁎^	−0.09^⁎⁎⁎^
Computer experience					0.2^⁎⁎⁎^	0.15^⁎⁎⁎^	0.05^⁎⁎⁎^	0.06^⁎⁎⁎^	0.06^⁎⁎⁎^	0.18^⁎⁎⁎^
Computers at home						0.2^⁎⁎⁎^	0.09^⁎⁎⁎^	0.09^⁎⁎⁎^	0.06^⁎⁎⁎^	0.24^⁎⁎⁎^
Parent with higher education							0.06^⁎⁎⁎^	0.09^⁎⁎⁎^	0.06^⁎⁎⁎^	0.23^⁎⁎⁎^
Time drop								0.34^⁎⁎⁎^	0.14^⁎⁎⁎^	0.53^⁎⁎⁎^
Performance drop									0.12^⁎⁎⁎^	0.39^⁎⁎⁎^
Response rate										0.28^⁎⁎⁎^
CIL test score										

^∗∗∗^*p* < 0.001; ^∗∗^*p* < 0.01; ^∗^*p* < 0.05

## 4 Methods

We use linear models in which CIL plausible values are regressed against the independent variables identified in Section 3. Recall that the hypotheses we wish to test are that our proxies for non-cognitive skills act towards narrowing the CIL gaps along gender and socioeconomic status lines, while they work in the opposite direction as regards the immigrant background gap. Testing these hypotheses requires a comparison of a “baseline” model, which doesn't include proxies for non-cognitive skills, with an “extended” model where our proxies for non-cognitive skills are added. If such hypotheses are correct, then the regression coefficients for gender and socioeconomic status will be statistically significantly smaller in the extended model than in the baseline model, whereas the opposite will occur for the coefficient on immigrant background.^6^

It is reasonable to assume that some part of the variability in CIL scores can be attributed to systematic differences between schools in terms of ICT-related resources and policies. That is, some schools are likely to give their students more opportunities than others to develop their digital skills. It is of paramount importance to account for the influence of these factors as they are likely to be related to some of the variables that are of interest in this study. For example, if students from more advantaged backgrounds are more likely to end up in schools better endowed with IT resources than students from less advantaged backgrounds, then the coefficient on socioeconomic status will be picking up the effect of the between-school differences in ICT resources. Thus, unless accounted for, the between-school variability may result in an upwardly biased estimate of the coefficient on socioeconomic status.

To handle this problem, we mean-center all our variables at the school-level. This way, we are able to remove the part of the variability in CIL test scores that is due to schools — and also, indirectly, the one due to countries/regions/cities — leaving only the part that can be attributed to differences across individual students (Enders & Tofighi, 2007). In other words, both the between-school and between-country/region/city variances of the mean-centred variables are zero.^7^

Since the dataset used in this paper is organised along three levels, with students nested in schools and schools nested in countries/regions/cities, a multilevel framework could, in principle, be used in the analysis of ICILS 2018. However, given that the data come from a complex, multistage survey, they have to be properly weighted at each level of the analysis to obtain unbiased estimates of the relevant parameters. Unfortunately, while survey weights for students and schools are provided in the ICILS 2018 dataset, this does not occur for countries/regions/cities. This ultimately reflects the fact that countries/regions/cities were not randomly selected from an underlying population. One therefore faces a choice between (*a*) fitting a three-level model to the pooled data discarding the survey weights altogether and (*b*) fitting a two-level model with survey weights for students and schools for each of the participating countries/regions/cities separately. In the former case, however, the resulting parameter estimates are likely to be biased. In the latter case, in turn, an additional difficulty arises: a rather high rate of missing data at the school level. The fixed-effects approach, by contrast, allows one to run the analysis on the pooled dataset using only student-level data (including the survey weights). This way, one can obtain unbiased estimates of the parameters of interest while controlling for time-invariant characteristics, observed and unobserved, of schools and countries/regions/cities (see Jæger, 2011, for an example of a similar approach).

All the regressions reported in the next section are estimated using the mean-centred variables. As a result, the intercept in all the models is equal to 0. However, the slope coefficients have the same interpretation they would have in a standard OLS model. That is, they can be interpreted in terms of a change in the CIL test score associated with a 1-unit change in the given independent variable when all other independent variables are held constant. Because ICILS is based on a complex sampling design, point estimates of the model parameters are obtained by applying survey design weights. Standard errors of the estimates, in turn, are obtained using replicate weights included in the ICILS dataset (for details concerning the sample design and how to use the weights in the analyses of ICILS data, see Mikheeva & Meyer, 2020). All the regression models are estimated using functions in the R package survey (Lumley, 2010, Lumley, 2020). Note that the standard errors returned by the package are heteroskedasticity-consistent by default.

## 5 Results

To verify whether our proxies for non-cognitive skills vary systematically by gender, socioeconomic status, and immigrant background, we regressed these proxies against the set of independent variables described earlier. The results, which are presented in Table 3, show that the coefficient on gender is consistently positive and statistically significant, confirming that female students outperform male students in each of the non-cognitive skills. As for socioeconomic status, its coefficient is positive across all three models, as expected. However, it fails to reach statistical significance in the model for item response rate. Finally, against our expectations, the coefficient on immigrant background is negative, suggesting that migrant students tend to have lower levels of the relevant non-cognitive skills than their native-born peers. The fact that the difference in non-cognitive skills by immigrant background is in the opposite direction of what we expected has important implications for our Hypothesis 3. We will return to this issue in the next Section.

**Table 3:** Results from for fixed-effect linear models for the three proxies for non-cognitive skills.

	Time drop	Performance drop	Item response rate
Female	11.34^⁎⁎⁎^	0.02^⁎⁎⁎^	0.01^⁎⁎⁎^
	(0.80)	(0.00)	(0.00)
	*0*.*018*	*0*.*012*	*0*.*003*
Age	−5.21^⁎⁎⁎^	−0.01^⁎⁎⁎^	−0.01^⁎⁎^
	(0.88)	(0.00)	(0.00)
	*0*.*003*	*0*.*002*	*0*.*001*
Migrant	−3.96^⁎⁎^	−0.01^⁎⁎^	−0.01^⁎^
	(1.39)	(0.00)	(0.00)
	*0*.*001*	*0*.*001*	*0*.*000*
Computers at home	1.29	0.01^⁎⁎^	0.01^⁎⁎^
	(0.89)	(0.00)	(0.00)
	*0*.*000*	*0*.*000*	*0*.*001*
Computer experience: [1, 3)	5.83^⁎⁎⁎^	0.01^⁎^	0.01
	(1.27)	(0.00)	(0.00)
	*0*.*001*	*0*.*000*	*0*.*000*
Computer experience: [3, 5)	6.73^⁎⁎⁎^	0.01^⁎⁎⁎^	0.01^⁎^
	(1.55)	(0.00)	(0.00)
	*0*.*002*	*0*.*001*	*0*.*001*
Computer experience: [5, 7)	9.36^⁎⁎⁎^	0.02^⁎⁎⁎^	0.01^⁎^
	(1.33)	(0.00)	(0.00)
	*0*.*003*	*0*.*001*	*0*.*001*
Computer experience: 7+	9.30^⁎⁎⁎^	0.01^⁎⁎⁎^	0.02^⁎⁎⁎^
	(1.29)	(0.00)	(0.00)
	*0*.*003*	*0*.*001*	*0*.*002*
Parents with higher education	3.72^⁎⁎⁎^	0.01^⁎⁎^	0.001
	(0.90)	(0.00)	(0.00)
	*0*.*001*	*0*.*001*	*0*.*000*
*N*	40,403	40,403	40,403
*R*^2^	0.03	0.02	0.01
Adj. *R*^2^	−0.11	−0.12	−0.13

^∗∗∗^*p* < 0.001; ^∗∗^*p* < 0.01; ^∗^*p* < 0.05

Values in italic font indicate effect sizes for the regression coefficients.

Along with the regression coefficients, Table 3 provides (in italics) values of partial (Cohen's) *f*^2^, a common measure of effect size (Selya et al., 2012). The *f*^2^ values of 0.35, 0.15, and 0.02 are used as thresholds for large, medium, and small effect sizes, respectively. Compared to these thresholds, the regression coefficients in Table 3, even if statistically significant, have very small effect sizes. Overall, the independent variables account for only a small part of the variability in our proxies for non-cognitive skills, which is further confirmed by low values of *R*^2^ and the adjusted *R*^2^ (note that, unlike *R*^2^ which is always bounded between 0 and 1, adjusted *R*^2^ can assume negative values).

Moving on to the main focus of our analysis, Table 4 presents results for another set of regression models. Model 1 is our baseline model. In Models 2 through 4 we add our proxies for non-cognitive skills, one at a time. In Model 5, all three proxies are included together. Using VIF to check for multicollinearity, we find that all variables included in our models have VIF values well below 10, which means that there is no strong multicollinearity among the variables considered (see Gujarati, 2004, p. 362).

**Table 4:** Results from the fixed-effects linear models for the CIL test scores.

	Model 1	Model 2	Model 3	Model 4	Model 5
Female	16.91^⁎⁎⁎^	8.01^⁎⁎⁎^	12.30^⁎⁎⁎^	15.19^⁎⁎⁎^	5.39^⁎⁎⁎^
	(1.37)	(1.30)	(1.36)	(1.31)	(1.27)
	*0*.*016*	*0*.*005*	*0*.*009*	*0*.*013*	*0*.*002*
Age	−16.30^⁎⁎⁎^	−12.21^⁎⁎⁎^	−14.39^⁎⁎⁎^	−15.16^⁎⁎⁎^	−10.86^⁎⁎⁎^
	(1.46)	(1.30)	(1.41)	(1.39)	(1.22)
	*0*.*011*	*0*.*008*	*0*.*010*	*0*.*011*	*0*.*007*
Migrant	−14.05^⁎⁎⁎^	−10.94^⁎⁎⁎^	−12.11^⁎⁎⁎^	−12.74^⁎⁎⁎^	−9.31^⁎⁎⁎^
	(2.41)	(2.06)	(2.24)	(2.35)	(1.97)
	*0*.*003*	*0*.*002*	*0*.*002*	*0*.*002*	*0*.*002*
Computers at home	11.97^⁎⁎⁎^	10.96^⁎⁎⁎^	10.90^⁎⁎⁎^	11.07^⁎⁎⁎^	9.82^⁎⁎⁎^
	(1.31)	(1.18)	(1.19)	(1.27)	(1.12)
	*0*.*006*	*0*.*006*	*0*.*005*	*0*.*005*	*0*.*006*
Computer experience: [1, 3)	21.26^⁎⁎⁎^	16.68^⁎⁎⁎^	19.70^⁎⁎⁎^	20.44^⁎⁎⁎^	15.84^⁎⁎⁎^
	(2.53)	(2.35)	(2.40)	(2.51)	(2.27)
	*0*.*006*	*0*.*005*	*0*.*006*	*0*.*006*	*0*.*005*
Computer experience: [3, 5)	33.03^⁎⁎⁎^	27.75^⁎⁎⁎^	30.41^⁎⁎⁎^	31.39^⁎⁎⁎^	25.78^⁎⁎⁎^
	(2.83)	(2.61)	(2.63)	(2.79)	(2.54)
	*0*.*015*	*0*.*014*	*0*.*014*	*0*.*014*	*0*.*013*
Computer experience: [5, 7)	41.06^⁎⁎⁎^	33.72^⁎⁎⁎^	37.78^⁎⁎⁎^	39.38^⁎⁎⁎^	31.66^⁎⁎⁎^
	(2.69)	(2.40)	(2.47)	(2.74)	(2.31)
	*0*.*022*	*0*.*019*	*0*.*020*	*0*.*021*	*0*.*018*
Computer experience: 7+	44.68^⁎⁎⁎^	37.38^⁎⁎⁎^	41.69^⁎⁎⁎^	42.07^⁎⁎⁎^	34.77^⁎⁎⁎^
	(2.51)	(2.25)	(2.33)	(2.40)	(2.09)
	*0*.*028*	*0*.*025*	*0*.*027*	*0*.*026*	*0*.*024*
Parents with higher education	12.12^⁎⁎⁎^	9.20^⁎⁎⁎^	10.98^⁎⁎⁎^	11.93^⁎⁎⁎^	8.83^⁎⁎⁎^
	(1.36)	(1.17)	(1.27)	(1.30)	(1.12)
	*0*.*006*	*0*.*005*	*0*.*006*	*0*.*006*	*0*.*005*
Time drop		0.78^⁎⁎⁎^			0.67^⁎⁎⁎^
		(0.02)			(0.02)
		*0*.*321*			*0*.*234*
Performance drop			208.56^⁎⁎⁎^		117.65^⁎⁎⁎^
			(5.29)		(5.23)
			*0*.*109*		*0*.*041*
Item response rate				157.70^⁎⁎⁎^	119.17^⁎⁎⁎^
				(6.61)	(6.14)
				*0*.*060*	*0*.*046*
*N*	40,403	40,403	40,403	40,403	40,403
*R*^2^	0.09	0.31	0.18	0.14	0.37
Adj. *R*^2^	−0.04	0.20	0.05	0.00	0.24

^∗∗∗^*p* < 0.001.; ^∗∗^*p* < 0.01.; ^∗^*p* < 0.05

Values in italic font indicate effect sizes for the regression coefficients.

The inclusion of the proxies for non-cognitive skills considerably improves the explanatory power of the model, as indicated by the values of the adjusted *R*^2^ in Table 4. While drop in response time turns out to have the largest effect on the adjusted *R*^2^, item response rate is found to have the lowest impact. This could be attributed to the relatively small variance of this variable in the ICILS 2018 sample.^8^

The signs of the coefficients on the proxies for non-cognitive skills are consistent with our expectations. Since drop in response time refers to a change in response time between the last and the first task, a value of −60 means that a student takes 60 s less to answer the last question in the test in comparison with the first one. On the other hand, a value of 30 indicates that a student takes 30 s more to solve the last task than the first one. Thus, following the interpretation of response time offered in previous studies, the former student is seen as less able to stay focused or lacking in grit than the latter. According to the estimates in Table 4, the difference in drop in response time between the two aforementioned students predicts a difference of [30 − (−60)] ⋅ 0.78 = 70.2 CIL points in Model 2 and of [30 − (−60)] ⋅ 0.67 = 60.3 CIL points in Model 5, holding the other independent variables constant.

Similarly, performance drop corresponds to a change in the probability of a correct answer between the last and the first question in the test. Thus, a value of −0.3 means that the probability of a correct answer drops by 30 % in the course of the test. By the same token, a value of 0.2 means that the probability goes up by 20 %. A student with the latter score is perceived to be more motivated and ambitious, more agreeable and conscientious and having a greater need for achievement than the former. The aforementioned difference in performance drop predicts a gap of [0.2 − (−0.3)] ⋅ 208.56 = 104.28 CIL points in Model 3 and of [0.2 − (−0.3)] ⋅ 117.65 = 58.83 CIL points in Model 5, keeping the other independent variables constant.

As for the item response rate, its coefficient can be interpreted as the expected difference in test scores between a student who answered all the questions in the background questionnaire and a student who answered none. For example, a student with a value of 0.9 answered 90 % of all the questions in the background questionnaire. Similarly, a person with a value of 0.75 answered three quarters of the questions. According the estimates reported in Table 4, this difference results in a gap of (0.9 − 0.75) ⋅ 157.7 = 23.66 CIL points in Model 4 and of (0.9 − 0.75) ⋅ 119.17 = 17.88 CIL points in Model 5, keeping the other independent variables constant.

As before, partial (Cohen's) *f*^2^ for each estimated coefficient is reported (in italics) in Table 4. For the three variables of interest — gender, socioeconomic status, and immigrant background, the effect sizes are pretty small, whereas for the proxies for non-cognitive skills they turn out to be small to medium, depending on the model specification, with the largest effect sizes observed for time drop. The small effect sizes should be a reason for caution when interpreting the regression coefficients, since, in spite of being statistically significant, they may not be “practically significant”. Such a result is not uncommon in studies using data from large-scale assessments in education, as shown in the online supplement to this paper.

**Fig. 1:**
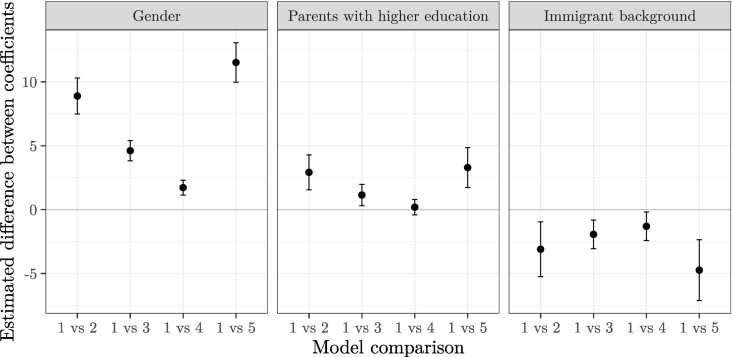
Differences in estimates of the coefficients on female, parents with tertiary education, and immigrant background and their corresponding 95 % confidence intervals across the models shown in Table 4.

Fig. 1 presents the results of the tests of our hypotheses. For each characteristic of interest, we compare its coefficient in the baseline model (Model 1) with the corresponding coefficient in the extended models (Models 2 through 5). Labels on the horizontal axis indicate the models that are being compared. The grey dots in Fig. 1 represent point estimates of the difference between the relevant regression coefficients in the models under comparison, whereas the vertical segments represent 95 % confidence intervals around the difference. Finally, the horizontal line at 0 represents the null hypothesis of no difference in the value of the coefficients in the two models under comparison. The coefficient on gender in the baseline model equals 16.91 (i.e., female students outperform male students by about 17 % of the standard deviation). However, adding drop in response time reduces the gender score gap to 8.01, a difference of nearly 9 points that is statistically significantly different from 0 at the level of *p* = 0.05 (see left panel of Fig. 1, the comparison of Models 1 and 2). When performance drop is added to the model, the estimated regression coefficient on gender is equal to 12.30, a difference of 4.61 points relative to the baseline model. Again, this difference is statistically significant. A similar observation can be made regarding the comparison of the gender coefficients in Models 1 and 4, although the difference in this case is rather small and, while statistically significant, has little practical meaning. Finally, the gender coefficient in Model 5 (where all the three proxies for non-cognitive skills are simultaneously included) is found to be equal to 5.39, a difference of more than 10 points relative to the baseline model, implying that a considerable part of the gender gap in the CIL test scores can be attributed to differences between male and female students in terms of non-cognitive skills. All in all, all our proxies turn out to narrow the gender gap in CIL scores, thus lending support to Hypothesis 1.

A similar pattern is observed as regards the effect of our proxies for non-cognitive skills on the socioeconomic gap in digital competences. The results of our baseline model suggest that students who have at least one parent with higher education score 12.12 CIL points more than those whose parents don't have higher education. In Model 2 which, along with all the variables included in the baseline model, also includes time drop, the estimated coefficient on parental education is lower by 3 points, a statistically significant difference (see Fig. 1, the comparison of Models 1 and 2 in the middle panel). To sum up, adding the proxies for non-cognitive skills to the baseline model leads to a statistically significant reduction in the socioeconomic gap (see Fig. 1, the middle panel), thereby lending support to Hypothesis 2, in all models except for Model 4.^9^

Finally, students with an immigrant background are, on average, found to have a lower CIL test score than their otherwise comparable native peers. While in the baseline model the estimated difference between native and immigrant students is about 14 points, in the full model the corresponding figure is 9.31 points. Thus, including all the three proxies reduces the gap by a third. What is more, the difference in the estimated coefficient on migrant background is statistically significant for all model comparisons, which *is not* consistent with Hypothesis 3.

Coefficients on computer experience and the number of computers at home are in line with expectations. As for age, its coefficient is negative and statistically significant, implying that, all else being equal, older students score worse on the CIL test. This mirrors results from other studies, not limited to student populations (see Munger et al., 2021, for instance), in which age was also found to correlate negatively with digital skills.

## 6 Discussion and conclusion

Following an increasing emphasis worldwide on the need to constantly monitor and compare educational quality and student learning across countries, there has been an expansion of international large-scale assessments. A great deal of evidence (e.g., Breakspear, 2012; Lundahl & Serder, 2020) suggests that the outcomes of these tests are taken in great consideration by educational stakeholders including policymakers, parents, and teachers. However, some students may not perform to the best of their ability given that such tests have no impact on their grades or other academic pursuits. Importantly, there might be systematic differences in this respect not only across students within a country, but also across students from different countries. All this underscores the importance of accounting for non-cognitive skills (e.g., motivation, ambition, and conscientiousness) before any inference is made from the results of these tests, especially in an international comparison setting.

Building on the findings of previous studies showing that non-cognitive skills affect mathematics, science, and reading test scores (Borghans & Schils, 2018), we conclude that the same holds also for digital skills. Using data from ICILS 2018, we find that different non-self-reported measures acting as proxies for non-cognitive skills turn out to be significant predictors of student digital competences as measured by the CIL test scores. More specifically, drop in response time to test questions, drop in performance along the test and non-response rate in the background questionnaire are all related to students' CIL test scores. Drop in response time, an indicator for student motivation, ambition, conscientiousness and agreeableness, emerges as the strongest predictor of student achievement among our proxies for non-cognitive skills.

Additionally, the empirical findings point at differences in non-cognitive traits across gender, immigrant background and socioeconomic status. Females, natives and students from more advantaged backgrounds tend, on average, to exhibit higher non-cognitive skills (as captured by our three proxies) than males, immigrants and students from less advantaged backgrounds, respectively. Our result on gender is consistent with expectations because, as argued earlier, women tend to be more conscientious and more motivated than men in schoolwork. In line with this consideration, it is not surprising that previous studies (see, for instance, Balart & Oosterveen, 2019) argue that female students are more likely to review their answers before handing in a test, less likely to get stuck on a question for a long time, and more likely to verify that all questions have been answered. Similarly, in agreement with the results of several works, our finding confirms the positive association between socioeconomic background and non-cognitive skills. As reported in Section 2.2.2, children from wealthier families have more opportunities to become perseverant and to develop a positive attitude towards learning than their peers from poorer families. Following from this, our results indicate that the gender and socioeconomic gaps in digital skills both decrease after accounting for the influence of non-cognitive variables.

On the other hand, at variance with the existing literature, our estimates suggest that non-cognitive skills work towards widening the native-immigrant gap in digital skills. A possible explanation for this discrepancy is that previous studies on the relationship between immigrant background and non-cognitive skills rely on self-reported proxies for non-cognitive skills, whereas we employ an indicator that considers students' behaviour on the test. Although many immigrant students report high intrinsic learning motivation, there is the possibility that this is essentially the result of the pressure placed on them by public opinion or their parents. They may feel socially obliged to report this in order to avoid negative stereotypes (Suarez-Orozco et al., 2017). Alternatively, while a positive attitude towards education and higher engagement in learning are generally found to be associated with better academic performance, this relationship may be weaker or non-existent for some groups of students, such as those with an immigrant background — this is often referred as the so-called “engagement-achievement paradox” (Shernoff & Schmidt, 2008). One reason for it is that, though immigrant parents pass on their academic aspirations and expectations to their children, their ability to actually support and shape the learning of their offspring is limited by a number of obstacles. For instance, due to language barriers and lack of familiarity with the host educational system, they may experience a poor understanding or mistrust of school-related feedback and have poor confidence in their ability to support children's homework (Yamamoto & Holloway, 2010).

This paper shows that, to be informative, results from large-scale educational assessments in all domains (including digital skills) should account for the fact that test scores are affected by non-cognitive skills that considerably vary across students both within and across countries. A standard strategy to handle this issue should be agreed upon and only results adjusted for differences in non-cognitive skills should be used for cross-country comparisons and accountability purposes. Otherwise, as shown by this study, there is, for instance, the risk that the importance of gender in explaining digital performance differences across students may be overstated. Where possible, policymakers should base the adoption of relevant education policies on valid, reliable, and comparable findings.

### 6.1 Limitations

Our study is not free from limitations. First, the country/region/city coverage of the ICILS data is somewhat limited. Extending the number of participants is needed to improve the generalisability of our results.

Second, although, as mentioned in Section 1, cognitive and non-cognitive skills tend to capture different dimensions with separate effects on students' outcomes, one cannot completely rule out the possibility that our proxies for non-cognitive traits pick up the effect of some cognitive capacities. For example, while analysing the impact of item position effects on school test results, Nagy et al. (2018) find that decoding speed, which is a prototypical exemplar of cognitive capacities, is key to maintain a constant level of effort in an exam.

Third, differences between students in terms of digital skills are also likely to be related to differences across countries/regions/cities in terms, for instance, of ICT infrastructure and ICT educational programmes. Unfortunately, in our analysis we are unable to clearly identify these factors and their relative importance. The unobserved heterogeneity that may be present in fixed-effects models can be considered as a “black box” (Hill et al., 2020). Thus, future analyses using the ICILS data could benefit from explicitly modelling the effects of country/region/city-level characteristics on students' CIL test scores.

Furthermore, digitals skills are a broad and complex construct, comprising different dimensions and different types of abilities. For example, the ability to digitally edit a photograph and post it on a website requires a different skill-set than, say, writing a macro to automate calculations in a spreadsheet. Our analysis is limited in that it focuses on a generic, broad indicator of ICT competency. Additional research is needed to investigate if the findings of the present study hold also for more specific domains of digital skills.

Finally, our results about the impact of non-cognitive skills on ICT test scores cannot be given a causal interpretation: there are likely to be (additional) observable or unobservable factors affecting both ICT test scores and non-cognitive skills. In addition, our analysis does not shed any light on the mechanisms through which non-cognitive skills may affect ICT test scores.

## Declaration of competing interest

None.

## Appendix A Supplementary material

Supplementary material Image 1

## Footnotes

1 Of course, in addition to cognitive and non-cognitive skills, other determinants of standardised test scores include physical, mental, and contextual factors (Rozon, 2013).

2 Conscientiousness is a personality trait that reflects the tendency to be responsible, organised, hard-working, and to comply with norms and rules.

3 Grit is a personality trait, which is defined as passion and perseverance to achieve meaningful long-term goals.

4 Non-cognitive skills can be measured using a personality questionnaire designed to classify individuals on dimensions such as conscientiousness or self-control. Alternatively, they can be inferred from patterns of responses (e.g., the number of questions left unanswered) to a questionnaire or test which wasn't designed specifically to measure non-cognitive skills. In the former approach, the relevant indicators are “self-reported”, as they are based on information provided directly by students. In the latter approach, the indicators are “non-self-reported”, as they are extracted by a researcher. Since the ICILS questionnaire doesn't have any section on personality, we used the non-self-reported approach in this study.

5 While, in contrast to ICILS, PIAAC is a test with no time limitation, one can reasonably argue that time per question might be related to non-cognitive skills also in ICILS, where the fixed time that students have to complete the CIL test could affect how they respond to individual questions. For example, more motivated and focused test takers are aware that they cannot spend too much time on a single question if they want to do well on the test.

6 Testing our hypotheses amounts to testing if the difference between regression coefficients on a given variable from two different regression models is statistically significantly different from 0. Performing such a test requires that the standard error of the difference is calculated first. Calculating the standard error, however, is not trivial (Clogg et al., 1995; Mize et al., 2019). In this study, we take advantage of replicate weights to estimate the standard error of a difference between two regression coefficients.

7 A school-specific mean of a variable mean-centred the school level is equal to 0. This applies to all schools in a given country/region/city, so the average taken over all the schools within that country/region/city will also be equal to 0. Thus, since the mean-centred variable has a mean of 0 in each country/region/city, there is no between-country/region/city variance.

8 The distribution of this variable is highly skewed, with most students having values close to 1.

9 Note that all of the above conclusions hold when, instead of parental education, we use other proxies for students' socioeconomic status (the results are available upon request). This suggests that our findings are robust with respect to the variable used to capture socioeconomic status.
