# Supplementary material for: Non-cognitive skills and social gaps in digital skills: Evidence from ICILS 2018
Source: Learn Individ Differ. 2023 Feb;102:None. doi: 10.1016/j.lindif.2022.102254 (PMC10028727; doi:10.1016/j.lindif.2022.102254)
Supplement: Supplementary file 1 — Supplementary material [file mmc1.pdf]

Online supplement to article

Non-cognitive skills and social gaps in digital skills: Evidence from ICILS 2018

A comparison with the results from PISA 2018

In an attempt to compare the effect sizes for the coefficients on gender, socioeconomic status, and immigrant background in our study with estimates based on other datasets, we have estimated three fixed-effect models using data from the most recent cycle of OECD's *Programme for International Student Assessment* (hereafter, PISA 2018). In these models, students' scores in *reading*, *mathematics*, and *science* are regressed on a set of variables that are as similar to the ones we use in the main text as possible; these variables include:

- student's gender, coded 1 for women and 0 for men;
- student's age;
- student's immigrant background, coded 1 for native-born students and 0 for students with first-generation or second-generation immigrant background;
- having computers at home, coded 1 if a student reported having a desktop computer or a laptop at home and 0 otherwise,
- years of computer experience, represented by a set of dummy variables: no experience at all; up to 2 years of experience; 3 to 5 years of experience; 6 - 8 years of experience; and more than 8 years of experience,
- parents with higher education, coded 1 if a students has at least one parent with a higher education degree and 0 otherwise.

Questions concerning the presence of computers at home and the years of computer experience come from an *optional* ICT familiarity questionnaire, not from the main student questionnaire. Because of this, the analysis is limited to countries in which the optional questionnaire was administered. Out of 81 countries participating in PISA 2018, a total of 53 chose the ICT familiarity option. Note that the two computer-related variables in PISA 2018 are not identical to those in ICILS 2018. For instance, students participating in PISA 2018 were asked to indicate which of the digital devices listed in the relevant question they had at home, whereas in ICILS 2018 they were asked explicitly how many of such devices they had at home. In the main text, we distinguish between students with at least two computers at home and the rest. Based on the PISA 2018 data, we can only distinguish between students who have at least one computer at home and the rest. Also, the question about years of

computer experience is worded differently in PISA 2018 and in ICILS 2018. In the former study, students were asked about the age at which they first started using digital devices. In the latter, they were asked explicitly about how many years in total they had been using digital devices. Assuming that students participating in PISA 2018 were 15 at the time of the survey, since 15-year-olds are the target population in PISA, students' responses to the computer experience question in PISA 2018 can be translated to the same "format" as in ICILS 2018. Note, also, that the categories of responses to that question differ across the two surveys. In spite of these differences, however, we believe that these variables are sufficiently similar for us to make comparisons between the results of the two analyses. Finally, before fitting the models to data from PISA 2018, we centered all variables at the school level, effectively removing the part of the variation that is due to schools and countries. The resulting models are equivalent to the baseline model (i.e., Model 1 in Table 3) in the main text: these models do not include any measures of non-cognitive skills similar to the ones we build in the paper.

To measure effect sizes of the coefficients on the variables included in our regression models, we use a measure that is known as Cohen's partial f^2. It is commonly used to capture effect sizes of coefficients in multiple regression. Its definition is quite straightforward (see Cohen 1988):

$$f^2 = \frac{R^2_{AB} - R^2_A}{1 - R^2_{AB}}$$

The formula above assumes that we compare two models: a "reduced" one, in which A is the only predictor and an "extended" one, in which there are two predictors, A and B, B being the variable of interest — i.e., the one whose effect size we wish to estimate. Obviously, the logic extends to more complex models which have more than 2 predictors. Thus, Cohen's partial f^2 for a given variable in a multiple regression model results from a comparison of the model with an otherwise similar model from which the focal variable is dropped. For instance, partial f^2 for age in the model for the performance in reading results from a comparison of this model with a reduced one from which age is dropped, while the other independent variables are kept. R^2 for these two models are fed to the above formula. The results are shown in Table 1. As in the main text, for each coefficient, we provide the estimate, its standard error (in brackets), and Cohen's partial f^2 (in italics).

Our primary interest is to see if the effect sizes for the coefficients on gender, socioeconomic status, and immigrant background in PISA 2018 are similar to those in ICILS 2018. As we can see, they are: the estimated effect sizes for all three characteristics of interest across all three models are very low. So, the very weak effect sizes are not typical just of ICILS, as they arise in other large-scale assessments, too.

	Reading	Mathematics	Science
Female	18.65^{***}	-11.40^{***}	-4.62^{***}
	(1.15)	(1.25)	(1.14)
	0.013	*0.006*	*0.001*
Age	12.03^{***}	10.78^{***}	9.80^{***}
	(2.23)	(2.31)	(2.22)
	0.002	*0.002*	*0.001*
Native-born	11.64^{***}	8.17^{**}	12.84^{***}
	(2.99)	(2.78)	(3.48)
	0.001	*0.001*	*0.002*
Computers at home	7.62^{***}	7.54^{***}	6.80^{***}
	(1.68)	(1.76)	(1.59)
	0.001	*0.001*	*0.001*
Computer experience: up to 2 years	33.49^{***}	26.56^{***}	28.14^{***}
	(4.60)	(6.49)	(4.17)
	0.002	*0.001*	*0.001*
Computer experience: 3 to 5 years	50.46^{***}	41.44^{***}	43.20^{***}
	(4.10)	(6.22)	(4.00)
	0.004	*0.003*	*0.003*
Computer experience: 6 to 8 years	65.10^{***}	54.57^{***}	54.69^{***}
	(4.02)	(6.04)	(4.06)
	0.006	*0.005*	*0.005*
Computer experience: more than 8 years	70.76^{***}	59.99^{***}	61.41^{***}
	(4.08)	(5.87)	(3.91)
	0.008	*0.007*	*0.006*
Parents with higher education	9.67^{***}	12.12^{***}	11.60^{***}
	(1.40)	(1.47)	(1.49)
	0.003	*0.006*	*0.005*
N	324, 988	324, 988	324, 988
R^2	0.04	0.04	0.03
Adj. R^2	−0.08	−0.08	−0.09

$^{***}p < 0.001$; $^{**}p < 0.01$; $^{*}p < 0.05$

Values in italic font indicate effect sizes for the regression coefficients

Table 1: Results from the fixed-effects linear models for test scores in PISA 2018

Table 2: Descriptive statistics

Variable	Statistic	CHL	DEU	DNK	DNW	FIN	FRA	ITA	KAZ	KOR	LUX	PRT	RMO	URY	USA
Female	Mean	0.51	0.50	0.50	0.49	0.51	0.51	0.48	0.49	0.49	0.49	0.49	0.50	0.50	0.51
	S.D	0.50	0.50	0.50	0.50	0.50	0.50	0.50	0.50	0.50	0.50	0.50	0.50	0.50	0.50
Age	Mean	14.11	14.39	14.89	14.37	14.80	13.80	13.25	14.33	14.21	14.48	13.97	14.77	14.35	14.19
	S.D	0.64	0.57	0.42	0.56	0.35	0.44	0.43	0.48	0.30	0.64	0.73	0.41	0.91	0.44
Immigrant background	Mean	0.05	0.23	0.09	0.26	0.03	0.13	0.10	0.09	0.00	0.49	0.06	0.09	0.02	0.05
	S.D	0.21	0.42	0.28	0.44	0.16	0.34	0.30	0.28	0.06	0.50	0.24	0.28	0.12	0.22
Computer experience: 1 - 3 years	Mean	0.26	0.23	0.18	0.24	0.10	0.22	0.23	0.23	0.21	0.24	0.11	0.09	0.20	0.19
	S.D	0.44	0.42	0.38	0.43	0.30	0.41	0.42	0.42	0.41	0.43	0.31	0.29	0.40	0.39
Computer experience 3 - 5 years	Mean	0.20	0.31	0.20	0.29	0.17	0.27	0.28	0.25	0.19	0.28	0.21	0.19	0.20	0.21
	S.D	0.40	0.46	0.40	0.45	0.38	0.44	0.45	0.43	0.39	0.45	0.41	0.39	0.40	0.41
Computer experience: 5 - 7 years	Mean	0.18	0.21	0.26	0.21	0.29	0.21	0.20	0.17	0.18	0.21	0.28	0.26	0.19	0.19
	S.D	0.39	0.41	0.44	0.41	0.45	0.40	0.40	0.38	0.39	0.41	0.45	0.44	0.39	0.39
Computer experience: 7+ years	Mean	0.25	0.14	0.33	0.14	0.41	0.23	0.17	0.16	0.32	0.17	0.34	0.40	0.26	0.33
	S.D	0.43	0.35	0.47	0.35	0.49	0.42	0.38	0.37	0.47	0.37	0.47	0.49	0.44	0.47
Computers at home	Mean	0.67	0.74	0.93	0.73	0.73	0.73	0.58	0.30	0.60	0.82	0.67	0.73	0.55	0.70
	S.D	0.47	0.44	0.26	0.44	0.44	0.45	0.49	0.46	0.49	0.38	0.47	0.44	0.50	0.46
Parental education	Mean	0.36	0.31	0.40	0.32	0.54	0.31	0.32	0.36	0.72	0.41	0.33	0.77	0.25	0.48
	S.D	0.48	0.46	0.49	0.46	0.50	0.46	0.47	0.48	0.45	0.49	0.47	0.42	0.43	0.50
Parental occupation	Mean	0.49	0.60	0.73	0.60	0.58	0.60	0.48	0.54	0.64	0.54	0.51	0.86	0.33	0.63
	S.D	0.50	0.49	0.44	0.49	0.49	0.49	0.50	0.50	0.48	0.50	0.50	0.35	0.47	0.48
Drop in response time	Mean	-8.07	6.99	-1.20	8.38	-8.47	4.29	-1.15	-31.55	-21.43	-9.20	-4.12	19.64	-26.75	-4.08
	S.D	48.05	48.07	45.24	47.80	50.97	45.18	47.00	42.53	52.65	50.06	45.98	39.16	49.38	45.53
Performance drop	Mean	0.04	0.05	0.06	0.05	0.05	0.04	0.03	-0.02	0.08	0.02	0.04	0.07	0.00	0.04
	S.D	0.11	0.11	0.11	0.11	0.11	0.11	0.11	0.10	0.12	0.11	0.11	0.10	0.11	0.10
Response rate	Mean	0.97	0.96	0.96	0.96	0.98	0.95	0.97	0.95	0.99	0.95	0.98	0.97	0.84	0.95
	S.D	0.08	0.09	0.07	0.09	0.07	0.10	0.08	0.10	0.04	0.12	0.05	0.05	0.22	0.11

References

Cohen, Jacob. 1988. *Statistical Power Analysis for the Behavioral Sciences.* Hillsdale, NJ: Lawrence Erlbaum Associates, Inc.
